# Impact of aging on animal models of Parkinson's disease

**DOI:** 10.3389/fnagi.2022.909273

**Published:** 2022-07-28

**Authors:** Ida Hyllen Klæstrup, Mie Kristine Just, Karina Lassen Holm, Aage Kristian Olsen Alstrup, Marina Romero-Ramos, Per Borghammer, Nathalie Van Den Berge

**Affiliations:** ^1^Department of Biomedicine, Aarhus University, Aarhus, Denmark; ^2^DANDRITE-Danish Research Institute of Translational Neuroscience, Nordic-EMBL Partnership for Molecular Medicine, Aarhus University, Aarhus, Denmark; ^3^Institute for Clinical Medicine, Aarhus University, Aarhus, Denmark; ^4^Nuclear Medicine and PET, Aarhus University Hospital, Aarhus, Denmark

**Keywords:** Alpha-synuclein (a-Synuclein), Parkinson's disease, gut-brain axis, aging, animal models, autonomic nervous system

## Abstract

Aging is the biggest risk factor for developing Parkinson's disease (PD), the second most common neurodegenerative disorder. Several animal models have been developed to explore the pathophysiology underlying neurodegeneration and the initiation and spread of alpha-synuclein-related PD pathology, and to investigate biomarkers and therapeutic strategies. However, bench-to-bedside translation of preclinical findings remains suboptimal and successful disease-modifying treatments remain to be discovered. Despite aging being the main risk factor for developing idiopathic PD, most studies employ young animals in their experimental set-up, hereby ignoring age-related cellular and molecular mechanisms at play. Consequently, studies in young animals may not be an accurate reflection of human PD, limiting translational outcomes. Recently, it has been shown that aged animals in PD research demonstrate a higher susceptibility to developing pathology and neurodegeneration, and present with a more disseminated and accelerated disease course, compared to young animals. Here we review recent advances in the investigation of the role of aging in preclinical PD research, including challenges related to aged animal models that are limiting widespread use. Overall, current findings indicate that the use of aged animals may be required to account for age-related interactions in PD pathophysiology. Thus, although the use of older animals has disadvantages, a model that better represents clinical disease within the elderly would be more beneficial in the long run, as it will increase translational value and minimize the risk of therapies failing during clinical studies. Furthermore, we provide recommendations to manage the challenges related to aged animal models.

## Introduction

Parkinson's disease (PD) is an age-dependent neurodegenerative disorder that affects 1–1.8% of people above 65 years old and 2.6–3% of people above 80 years old (Wirdefeldt et al., 2011; Pringsheim et al., 2014). PD is characterized by the presence of alpha-synuclein (asyn) pathology and neurodegeneration across the central and peripheral nervous system (CNS and PNS) (Beach et al., 2010), causing a wide range of motor and non-motor symptoms (Borghammer, 2018; Skjærbæk et al., 2021). Aging is the greatest risk factor of PD, as reflected in the increasing prevalence with aging. However, PD etiology is multi-factorial as a combination of many variables, including disease onset site (Borghammer and Van Den Berge, 2019; Horsager et al., 2020), brain and autonomic connectome (Borghammer et al., 2021a), and other factors such as cellular vulnerability (Alegre-Abarrategui et al., 2019), mitochondrial dysfunction (Chen et al., 2019), oxidative stress (Dumitrescu et al., 2018), neuroinflammation (Tansey and Romero-Ramos, 2019), gut permeability (van IJzendoorn and Derkinderen, 2019), microbiome (Aho et al., 2019) and environmental factors, may also contribute to disease initiation and development. This mix of causative variables probably contributes to the highly heterogeneous clinical profile observed amongst patients.

Recently it has been postulated that PD may start in the brain (also called brain-first PD) or elsewhere in the body, usually the gut (also called body-first PD), and that the disease initiation site is associated with a certain clinical manifestation of disease, namely autonomic dysfunction prior to dopamine deficit and more symmetric parkinsonism in body-first PD, and vice versa in brain-first PD (Borghammer and Van Den Berge, 2019; Horsager et al., 2020; Borghammer et al., 2021a; Knudsen et al., 2021). Importantly, neuropathological evidence indicates at least 50% prevalence of body-first PD (Borghammer et al., 2021b). It is thought that brain-first PD is mostly clinically silent until motor symptoms emerge, which makes pre-motor diagnostics challenging in this subtype, and which may require the discovery of an asyn positron emission tomography (PET) tracer. On the other hand, early identification of body-first PD has become feasible in the pre-motor phase by using a combination of several biomarkers, including the detection of peripheral pathology in skin and gut biopsies, the presence of idiopathic REM sleep behavior disorder (RBD), as well as cardiac and/or enteric denervation measured with MIBG scintigraphy or donepezil PET, respectively (Horsager et al., 2020). Despite the high prevalence of body-first PD and the opportunity to diagnose and intervene early (prior to extensive damage in the brain), preclinical research aiming to find a cure has almost exclusively been using brain-first PD models (Van Den Berge and Ulusoy, 2022).

It is estimated that PD will affect 17.5 million people worldwide by 2040, due to aging of the population, increased longevity, reduced smoking and increasing industrialization (Dorsey et al., 2018). Moreover, recent studies have linked COVID-19 to neurodegenerative disorders, particular in COVID-19-patients that experienced encephalitis (Brundin et al., 2020; Shen et al., 2022), and it has been reported that COVID-19 may accelerate brain aging (Mavrikaki et al., 2021). Thus, it is possible that there will be even more people affected with PD than anticipated. Research in animal models has yielded promising disease-modifying treatment targets and strategies, including immunotherapy (Folke et al., 2022). Unfortunately, translational research and clinical trials have generated disappointing results, indicating that the models currently used in preclinical research may be suboptimal. This is especially the case in the field of neuroscience, and therefore there is a need to rethink factors of importance for increasing the value of preclinical experiments (Alstrup and Sonne, 2019). We believe that the limited translational outcome could be attributed to inadequate modeling of human disease by excluding trivial disease factors such as (1) peripheral pathology and autonomic dysfunction, and (2) old age.

Although it is well-known that age is the highest risk factor in developing PD, the use of aged animals in preclinical research is limited, due to experimental, time- and cost-related challenges. Recent *in vivo* experimental studies, implicate the role of age as a facilitator for PD pathology development and neurodegeneration across different PD animal models. The increased vulnerability of aged animals to develop disease, indicate a compromised validity of findings from studies using young animals, particularly concerning translational studies focused on biomarker and treatment validation. More specifically, the effect of aging on disease-modifying treatment should be taken into account in experimental treatment validation, as aged PD animal models display reduced therapeutic response compared to young PD rodents in some studies (Sortwell et al., 2001; Grimmig et al., 2018).

Moreover, aging has been reported to promote gut permeability and inflammation (Thevaranjan et al., 2017; Ticinesi et al., 2019), possibly contributing to the large prevalence of body-first PD. Here, we aim to review current findings on the role of aging on pathology and neurodegeneration in body-first and brain-first PD models. The cause of progressive degeneration in PD remains to be elucidated, albite different studies have suggested that both brain and peripheral inflammation could play a key role in disease progression. Hence, we also include the role of inflammation in the aging brain and body in this review. Finding a cure for PD may be accelerated if future treatment identification/validation studies take a more comprehensive approach and use animal models that include several features of human PD, incl. peripheral pathology and old age.

## Impact of aging on pathology and neurodegeneration

### PD subtypes

Since the discovery that motor symptoms in PD are associated with loss of dopaminergic neurons in the substantia nigra (SN) (in the 1960s), and later also with the presence of pathogenic asyn in the brain (in 1997), animal models have been focused mainly on modeling disease in the CNS. The classical motor types are (1) tremor-dominant (benign), (2) postural-instability gait disorder, and (3) a mixed form (malignant). Despite thorough investigation of the different motor phenotypes, the underlying disease mechanisms underlying these subtypes are still unknown, making them difficult to model (Ren et al., 2020; von Coelln et al., 2021).

Modeling of CNS pathology has been pursued using different disease modeling strategies: (1) by artificial induction of dopamine deficit through delivery of a neurotoxin, (2) by viral-mediated local overexpression of asyn, (3) through induction of artificial asyn pathology by delivery of different aggregated forms of asyn (also called fibrils) in the brain, or (4) by using transgenic models characterized by elevated human asyn expression under specific promoters of wide or limited expression in the rodent (Dawson et al., 2018; Huntington and Srinivasan, 2021). CNS aggregate pathology has also been observed in other less common transgenic models, such as those that aim to induce PD by elimination of mitochondrial function (e.g., MitoPark) (Ekstrand and Galter, 2009). Often a combination of models is used to increase disease severity. Animal studies that focus on modeling the CNS by means of an intracerebral insult are referred to as brain-first PD models, since they model human brain-first PD with CNS involvement prior to autonomic involvement and asymmetric motor deficit upon unilateral lesioning (Van Den Berge and Ulusoy, 2022).

In 2003, Braak reported that PD pathology seems to progress in a predictable fashion from the dorsal motor nucleus of the vagus nerve (DMV) to the locus coeruleus (LC) at early disease stages, further involving the SN at mid disease stages, ultimately involving the entire cerebral hemispheres at late disease stages (Braak et al., 2003b). Braak hypothesized that a foreign pathogen may penetrate the gut epithelium where it initiates the formation of pathogenic asyn, and consequently spreads to the DMV via the vagus nerve (Braak et al., 2003a), also called the gut- or body-first hypothesis. Since then, multiple studies have investigated the feasibility of induction of pathology in the gut (but also in other peripheral organs such as the muscle or autonomic ganglia) with subsequent propagation of pathology to the brain via the vagus nerve and also non-vagal circuits (Van Den Berge and Ulusoy, 2022). Animal models focusing on modeling of PNS and CNS pathology by disease initiation in the gut (or other peripheral organs) are referred to as body-first PD models, as they aim to recapitulate human body-first PD with autonomic involvement prior to CNS involvement and more symmetric dopamine deficit. Details about brain- and body-first PD subtypes were reviewed previously (Borghammer and Van Den Berge, 2019); Borghammer et al., 2021a). These hypothesized disease subtypes are based on imaging findings in patients (Horsager et al., 2020; Knudsen et al., 2021) and neuropathological evidence from large patient cohorts (Beach et al., 2009; Raunio et al., 2019; Borghammer et al., 2021b). A complete review on brain-first and body-first PD animal models was published recently (Van Den Berge and Ulusoy, 2022). Below, we list the studies investigating the direct effect of old age on PD development (including but not limited to the presence of asyn pathology, neurodegeneration and/or symptoms) in brain-first and body-first PD animal models. Note, direct assessment of aging requires artificial disease initiation at old age, and not long-term follow-up upon PD initiation at young age.

### Impact of aging in brain-first PD

It is well-known that motor performance and the brain's dopaminergic system decline with age (Seidler et al., 2010; Hoogendam et al., 2014). Several age-dependent changes, including changes in dopamine metabolism, uptake and synthesis, receptor sensitivity, mitochondrial function, receptor sensitivity, calcium dynamics, iron concentration, and proteostatic function make dopaminergic neurons of the nigrostriatal pathway more susceptible to senescence-driven dysfunction compared to other neuronal populations in the CNS (Reeve et al., 2014; Collier et al., 2017; Zucca et al., 2017; Surmeier, 2018). Despite decades of research with over 1,500 studies investigating dopaminergic pathways and the effect of dopamine deficit in the brain, only a few studies have evaluated the effect of aging on CNS sensitivity to neurodegeneration, pathology and motor deficit. Such studies artificially induced dopamine depletion with a neurotoxin (6-OHDA or MPTP) or viral vector-mediated delivery of asyn and are listed in Table 1.

**Table 1:** Effect of aging in brain-first PD animal models.

**References**	**Strain/stock**	**Inoculation** **(type, site, dose)**	**CNS findings**
**6-OHDA**			
Ricaurte et al. (1988)	2-, 8–12-, and 18–24-month old C57BI/6J mice	6-OHDA, ventricle, 25 or 50 μg	6-OHDA-induced dopamine depletion in the striatum (HPLC) is dose-, but not age-dependent
Ling et al. (2000)	4-, 12-, 18-, and 23-month old Fisher 344 rats	6-OHDA, mesostriatal fiber bundle and SN, 10 μg	- Age-dependent decrease in basal DA content of the striatum - Age-dependent decrease in striatal trophic activity - Age-dependent increase in dopamine depletion
Cass et al. (2002, 2005)	4–5-, 13–14-, and 24–25-month old Fischer-344 rats	6-OHDA, ventricle, 50 or 100 μg	- Enhanced nigrostriatal DA lesion in aged compared to younger rats, quantified by dopamine release and uptake using electrochemical recordings, and in post-mortem tissue levels of dopamine using HPLC - Spontaneous locomotor activity (in dedicated activity chamber) was decreased in middle-aged and aged rats compared to young rats
Tamás et al. (2005, 2006)	3- and 18–20-month old Wistar rats	6-OHDA, SN, 8 μg	Slightly higher loss of TH-positive neurons in the SN of aged compared to young rats and more severe motor dysfunction (open field test) in aged rats
Villar-Cheda et al. (2012)	Young and aged rats	6-OHDA, SN	- Increased activation of NADPH oxidase complex and increased levels of the proinflammatory cytokines in the SN - Increased 6-OHDA-induced dopamine depletion in aged rats
Barata-Antunes et al. (2020)	10-weeks and 17-month old Wistar–Han rats	6-OHDA, medial forebrain bundle„ 8 μg	- Age-dependent neurodegeneration in ipsilateral SNpc (TH) and age-dependent decline of skilled motor function (staircase test) - No age-dependent differences in striatal fiber density (TH) and non-skilled motor performance (toxin-induced rotation behavior and cylinder test)
**MPTP**			
Ali et al. (1993)	23-days, 7- and 12-month old C57/B6N mice	MPTP, i.p 4 times every 2 h, 10 mg/kg MPTP	- Slight decrease of striatal dopamine and its metabolites (DOPAC and HVA) 24 h post dosing in 23-day old mice - 50–65% decrease of striatal dopamine and metabolites in 7-month old mice - 80% decrease of striatal dopamine and 60–80% decrease of DOPAC and HVA in 1-year old mice
Ovadia et al. (1995)	5–9-, 10–19-, and 20-23-year old rhesus monkeys	MPTP, carotid artery, 0.4–1.2 mg/kg, repeated injections until PD-like symptoms	-Age-dependent MPTP dose needed to produce stable, moderate PD-like symptoms: young monkeys required 3 times more MPTP compared to monkeys from 16-years old and above - Aged monkeys showed more severe bradykinesia, upper limb rigidity, balance and gait abnormalities, despite lower MPTP-dose
Sugama et al. (2003)	3- and 9–12-month old C57BL/6 mice	MPTP, i.p.	-Age-dependent TH neuronal loss with 72% loss in old and 47% in young mice at 14 days - Age-dependent microglial activation surrounding DA neurons, peaking at 3–7 days and largely gone by 14 days in old mice, and peaking at 1 day and devoid at 3 days in young mice
Collier et al. (2005, 2007)	rhesus monkeys, young adult (8–9.5 years), middle-aged (15–17 years), aged (21–31 years)	MPTP, carotid artery, 3–4 mg	- Age-dependent decreased striatal BDNF and GDNF levels (ELISA) in the intact striatum in aged group only -age-dependent decline in dopamine and homovanillic acid in intact striatum from middle-aged group - Compensatory increase in dopamine activity in lesioned striatum absent in aged group - Age-dependent morphological changes, including decreased striatal fiber density (TH), decreased nigral soma size, and optical density of TH, but no significant loss of neurons. - MPTP-induced increase in trophic activity that was sustained for at least 3 months in young monkeys, not sustained in middle-aged and aged monkeys - GDNF levels were unchanged with aging and at 3 months after dopamine depletion
Boger et al. (2008)	26-month old GFRα1 ± mice and WT littermates	MPTP, i.p. daily for 4 days, 20 mg/kg	GFRα1 ± mice show greater inflammation in the SN, and reduced striatal TH density compared to WT littermates, MPTP exacerbated these findings
Bourdenx et al. (2015)	2-month old C57Bl/6J, SAMP8 and SAMR1 mice	MPTP, i.p. daily for 5 days, 30 mg/kg	MPTP-induced nigrostriatal neurodegeneration in C57Bl/6 J but not in SAMP8 and SAMR1 mice at 21 days
**AAV-mediated asyn overexpression**			
Bourdenx et al. (2015)	2-month old C57Bl/6J, SAMP8 and SAMR1 mice, 2-year 6-year old marmoset monkeys	mice: SNc, AAV-h-asyn (120 nl−7.0 × 10^12^ vg/ml) monkeys: unilateral in SNc, AAV-h-asyn	- No difference in pathology, at 20 weeks post viral-mediated asyn overexpression, in WT and SAMP1/8 mice - Neurodegeneration in nigra in young WT mice, but not in old SAMP1/8 mice - No age-dependent difference in pathology at 11 weeks post viral-mediated asyn overexpression in monkeys

HPLC, high performance liquid chromatography; NADPH, nicotinamide adenine dinucleotide phosphate-oxidase; WT, wild-type; DA, dopaminergic; SNc, substantia nigra pars compacta; h-asyn, human asyn; i.p., intraperitoneal.

Aging of the dopaminergic pathways in the brain seems to increase the CNS's sensitivity to neurotoxins (Lotti, 2002; Phinney et al., 2006; Boger et al., 2008). This includes a larger nigrostriatal degeneration, a larger dopamine deficit and/or reduced motor function in older animals compared to young controls. However, some 6-OHDA-lesion studies did not observe significant differences in either striatal dopamine levels (Ricaurte et al., 1988) or nigrostriatal neurodegeneration (Tamás et al., 2005, 2006) in aged rats vs. young controls. Although old age did seem to affect 6-OHDA-induced motor dysfunction to some degree in these studies, which could be related to a higher degree of dopaminergic cell loss, the age-related inability of remaining neurons to compensate, or other neurochemical changes in old rodents (Tamás et al., 2006; Barata-Antunes et al., 2020). Interestingly, Tamás and colleagues found that both young and aging female rodents had significantly less dopaminergic cell loss upon 6-OHDA lesioning than males, in support of the higher prevalence of PD in men (Tamás et al., 2005, 2006).

In one study, MPTP did not cause a significant decline in TH-positive neurons in the SN of old monkeys, compared to young controls, but they did observe a significant reduction of optic density values in the SN as well as a reduction TH-positive fibers in the striatum of old monkeys (Collier et al., 2007). Note that these neurotoxin-lesion models mimic severe PD with rapid loss of dopaminergic neurons and pronounced motor deficits, depending on where the lesion is applied [medial forebrain bundle (MFB), striatum or SN]. For example, complete loss of striatal TH-positive fibers is achieved already 1 week post injection of 6-OHDA in the MFB, and does not worsen at later time points, whereas nigral degeneration continues to progress for some time, suggesting retrograde degeneration (Rentsch et al., 2019). The lack of age-dependent differences in striatal neurodegeneration in some studies is therefore not surprising, as sudden and massive lesioning does not allow investigation of the effect of age on disease progression.

However, the negative impact of old age on disease development in these neurotoxin models is still significant across studies, despite the severity of the induced lesion. It has been reported that young animals display increased levels of neurotrophic factors upon exposure to a neurotoxin. Neurotrophic factors are small extracellular proteins that support cell growth, differentiation, maturation, neuronal plasticity and survival, and are thus crucial for neuronal protection against injury, as well as for regeneration. There are four types of neurotrophic factors: (1) neurotrophins, including brain derived neurotrophic factor (BDNF); (2) glial cell-line derived neurotrophic factor ligands, including glial cell-line derived neurotrophic factor (GDNF) and neurturin (NRTN); (3) neuropoietic cytokines; and (4) mesencephalic astrocyte-derived neurotrophic factor (MANF) and cerebral dopamine neurotrophic factor (CDNF) (Yang and Gao, 2020). Neurotrophic factor alterations have been observed both in PD animal models and patients. Decreased neurotrophic factor levels, mainly BDNF and GDNF, have been reported in the SN, striatum and other central motor structures of PD patients. Importantly, the expression of the dopamine D3 receptor and tyrosine hydroxylase are mediated by BDNF, and decreased BDNF levels are correlated to the severity of dopaminergic neurodegeneration. Similarly, GDNF also supports motor and dopaminergic neuronal development and survival. And, marked reductions in GDNF levels have been observed in the SN of post mortem PD brains. Thus, deviations in these neurotrophic factors levels are detrimental to neuronal survival of dopaminergic neurons in PD (Sampaio et al., 2017). In neurotoxin models, BDNF, GDNF, and NRTN are the most commonly investigated. In aged animals, a reduced production of these trophic factors or an increased production of neuroinhibitory factors for dopaminergic neurons has been reported post lesion, compared to young animals (Ling et al., 2000; Yurek and Fletcher-Turner, 2000, 2001; Collier et al., 2005). These studies indicate that older animals are unable to produce a compensatory increase in neurotrophic factors to prevent toxin-induced neuronal death. Interestingly, one study showed that transgenic mice with reduced expression of the GDNF receptor, GFRα1, are more susceptible to age-related effects on the dopamine system, possibly due to increased inflammation, and that these effects were exacerbated post MPTP-lesion, supporting the importance of GDNF for maintaining the dopamine system during aging (Boger et al., 2008). Another study suggested that age-related microglial activation in the SN may contribute to the higher susceptibility to neurotoxin-induced neurodegeneration, with prolonged microglial activation surrounding dopaminergic neurons in aged mice (Sugama et al., 2003). Taken together, these findings indicate that aged animals have decreased compensatory mechanisms, including impaired plasticity and microglia, to deal with the administered neurotoxins.

Furthermore, the dopamine system is characterized by inherent compensation mechanisms for loss of dopamine function (including dopamine synthesis, release as well as dopamine receptor proliferation), as observed upon neurotoxic insult in young animals (Bezard and Gross, 1998). In aged animals, these compensation mechanisms seem to be lacking. One study reported an increased homovanillic acid-dopamine (HVA-DA) ratio in young and adult monkeys upon MPTP exposure. This compensatory increase in striatal dopamine signaling was absent in old monkeys (Collier et al., 2017). Another study reported an age-dependent decrease in basal dopamine (HVA-DA ratio) content of the striatum in aged rats. Importantly, these age-related changes in the dopamine system associated with changes in striatal neurotrophic activity. The authors suggest that the neurotrophic activity may contribute to the compensatory feedback process in order to maintain the cytoarchitectural integrity of the dopamine system (Ling et al., 2000).

Dopamine and noradrenaline neurotransmitter systems are reported to degenerate in parallel in prodromal body-first PD, and are associated with motor symptoms in PD (Andersen et al., 2020). Next to the dopamine system, increasing evidence associate other neurotransmitter deficiencies to PD. Acetylcholine (Bohnen and Albin, 2011) and serotonin (Huot et al., 2011; Grosch et al., 2016) contribute to motor and non-motor PD symptoms, as well as to levodopa-induced dyskinesia (Huot et al., 2011; Perez-Lloret and Barrantes, 2016). And, both the cholinergic (Schliebs and Arendt, 2011) and serotonergic (McEntee and Crook, 1991) systems seem to decline with aging as well. However, extensive investigations of these chemical systems in aged PD animal models are lacking. Furthermore, aging is associated with a natural decline in noradrenergic and dopaminergic neurons in the brain (Palmer and DeKosky, 1993; von Linstow et al., 2020). And, a lower dopaminergic cell count entails a higher risk to develop PD (von Linstow et al., 2020). These factors may also affect the outcome of neurotoxic insults since the amount of noradrenergic innervation has protective effects on dopaminergic neurons and motor function. Future preclinical studies should investigate how aging affects these pathways in aged PD animals, as such studies might reveal new treatment targets as well as improve treatment-induced complications in the elderly.

Furthermore, humans and monkeys exhibit increased levels of endogenous asyn with age (Chu and Kordower, 2007), possibly contributing to an increased risk of developing PD with age. In contrast, one study reported decreased levels of endogenous asyn in the SN of 20-month old wild-type mice (Mak et al., 2009). Bourdenx et al. investigated the effect of aging on asyn pathology and neurodegeneration in a viral-vector mediated model in young senescence-accelerated (SAMP8) and senescence-resistant (SAMR1) mice and 6-years-old marmoset monkeys. The authors reported no differences in pathogenic response to viral-mediated overexpression of asyn (nor to MPTP-insult). Importantly, nigral degeneration was only observed in young wild-type mice and not in SAMP8 or SAMR1 mice (Bourdenx et al., 2015), indicating a lack of susceptibility in the genetic background of SAMP8 and SAMR 1 mice, compared to wild-type mice. The SAMP8 strain is commonly used to investigate age-related cognitive decline in Alzheimer's disease (Liu et al., 2020), and thus, perhaps suboptimal to investigate PD pathology. In fact, another study also showed that the AKR/J genetic background in the SAMP8 and SAMR1 mouse lines is less sensitive to MPTP-induced neurotoxicity compared to wild-type C57Bl/6J mice (Hamre et al., 1999), although other unknown neuroprotective factors at play cannot be dismissed without further investigation. Therefore, results should be interpreted with caution regarding genetic background when working with transgenic mouse lines. It remains to be investigated whether aged wild-type mice would in fact show accelerated disease upon viral-mediated targeted overexpression of asyn. Second, the authors did not observe increased pathology development and nigral degeneration at 11 weeks post-viral-mediated overexpression of asyn in old, compared to young, monkeys (Bourdenx et al., 2015). It should be noted that 6-years old marmoset monkeys are not that old considering their 12-years lifespan. Since the prevalence of PD is highest above 80 years old, the 9.5-years equivalent in marmoset monkeys could have yielded a significant difference compared to young controls. These negative findings might indicate that very old wild-type animals are more suitable to mimic PD in the elderly, and that the use of mid-to-late adult animals is suboptimal to investigate pathological aging. Namely, two other studies using 20+ year old rhesus monkeys showed several age-related findings post-MPTP-lesion, including age-dependent motor dysfunction (Ovadia et al., 1995) and decreased striatal fiber density (Collier et al., 2007).

Importantly, differences in CNS-sensitivity to a neurotoxin (Song et al., 2015) or viral-mediated mediated overexpression of asyn (Huntington and Srinivasan, 2021) have been observed across different strains, even when not genetically engineered, and these differences might be magnified when working with aged animals. More studies in different strains need to be done to assess the effect of aging on multiple features of PD pathogenesis.

Due to the novelty of the model, there is not yet any study directly investigating the impact of old age on disease progression upon intracerebral injection of asyn fibrils. Most brain-seeding studies report time-dependent, progressive spread of asyn pathology throughout the CNS when following the animal from young to old age at multiple time-points post insult, hereby indirectly including age-related cellular and other mechanisms. However, direct evidence, meaning the insult is applied at old age, in brain-first seeding models is lacking. Our group has observed accelerated dopamine transporter deficit in old wild-type rats, compared to young, at 8.5 months post-injection of asyn fibrils in the amygdala (preliminary data).

Finally, there exists a wide range of studies using transgenic PD models that display increased vulnerability to develop PD-like pathology, neurodegeneration, as well as motor- and non-motor symptoms with increasing age (Dawson et al., 2010; Van Den Berge and Ulusoy, 2022). Although useful for investigating specific disease mechanisms, they are probably suboptimal to account for age-related cellular and other changes in idiopathic PD.

### Impact of aging in body-first PD

Pan-Montojo et al. (2010) showed that oral administration of the pesticide rotenone can cause local accumulation of pathogenic asyn in the gut, which subsequently can travel along parasympathetic and sympathetic nerves to the brain, reproducing Braak's stages of pathology. In 2014, Holmqvist et al. showed that fluorescence-tagged asyn was associated with a pathology able to travel from the gut to the DMV via the vagus nerve in rats in just 1 week (Holmqvist et al., 2014). These seminal studies provide the first preclinical evidence for Braak's gut-first hypothesis. Since then, more rodent studies have tried to recapitulate actual templating in the gut and subsequent gut-to-brain propagation of pathology, with varying results. Gut-to-brain or body-to-brain propagation of pathology can be modeled by injecting fibrils in the gut or in any other peripheral organ and could therefore be named body-first PD animal models. Interestingly, all studies that failed to induce robust gut-to-brain propagation used young wild-type animals, indicating additional cellular vulnerability is required such as transgenic overexpression of asyn or old age. Transgenic rodent models, characterized by elevated asyn expression, are more representative of genetic PD cases, exhibiting duplications or triplications of the SNCA gene. If the goal is to recapitulate human idiopathic PD (which represents ~90% of cases) one should use wild-type animals. However, only a few studies have investigated the direct effect of old age on disease progression in wild-type body-first PD models, listed in Table 2.

**Table 2:** Effect of aging in body-first PD animal models.

**References**	**Strain/stock**	**Inoculation** **(type, site, dose)**	**CNS findings**	**PNS findings**
**Rotenone**				
Phinney et al. (2006)	Young and old rats	Rotenone, systemic low dose	20–30% reduction of TH-positive neurons in the SN of aged but not young rats, increased glial cell activation in aged rats	N.A.
Pan-Montojo et al. (2010, 2012)	1-year old C57BL/6J mice	Rotenone, oral daily, 6 days/week, for 1.5, 2, 3, and 4 months, 5 mg/kg	- p-asyn in DMV and IML of rotenone-treated mice from 1.5 months - No cell loss in DMV and IML - Nigral dopaminergic degeneration and motor dysfunction (rotarod test) from 3 months	p-asyn and gliosis in ENS from 1.5 months, gut dysmotility from 2 months
Wang et al. (2015)	3- and 18- month old Sprague-Dawley rats	Rotenone, i.p. daily for 35 days, 0.5 mg/ml, at 1 ml/kg	- Motor dysfunction (rotarod and open field test) and striatal dopamine depletion in aged but not in young rats - Aging-related rotenone-induced increase in SIRT2 expression in the SN	N.A.
Almeida et al. (2016)	10 month old Lewis rats	Rotenone, s.c. daily for 4 weeks, 1 or 2 mg/kg/day	p-asyn in LC and SN in group with highest exposure	N.A.
Ureshino et al. (2018)	4–5 months-old (young) and 24–25 months-old (aged) Wistar rats	rotenone, s.c. daily for 12 days, 1.5 mg/kg/day	- Nigral dopaminergic degeneration, motor dysfunction (open field test), and elevated number of apoptotic nuclei in the striatum of aged and young treated rats - Swollen mitochondria in the striatum, and massive lipofuscin deposits in SN of aged rats.	N.A.
**Asyn fibrils**				
Challis et al. (2020)	2- and 16-month old C57BL/6N mice	Fibrils, duodenum, 18 μg	p-asyn accumulation in DMV of aged mice only, age-dependent striatal dopamine deficit	Disruptions of ENS network connectivity and the endoplasmic reticulum-Golgi-lysosome pathway
Van Den Berge et al. (2021)	3-, 12- and 18-month old Fischer 344 rats	Fibrils, duodenum, 30 μg	Brain-wide pathology (DMV/LC transient) in aged but not in young rats	- Pathology in heart, skin, muscle, kidney of old but not young - Synaptic dysfunction ENS and heart, incl. gastric dysfunction of old but not young

i.p., intraperitoneal; s.c., subcutaneous; WT, wild-type; TH, tyrosine hydroxylase; SN, substantia nigra; ENS, enteric nervous system; DMV, dorsal motor nucleus of the vagus nerve; LC, locus coeruleus; DSS, dextran sodium sulfate; GFAP, glial fibrillary acidic protein; p-asyn, phosphorylated asyn; N.A., not applicable.

The rotenone model, although strictly classified as a neurotoxin, is associated with asyn accumulation and pathology. Old age causes a more severe phenotype in rotenone-induced PD animal models with an increased decline in striatal dopamine and the antioxidant glutathione levels, with a concomitant increase of the marker of oxidative stress malondialdehyde in SN (Wang et al., 2015), increased dopaminergic cell death (Phinney et al., 2006; Ureshino et al., 2018), increased hyperphosphorylated asyn pathology (Pan-Montojo et al., 2010, 2012; Almeida et al., 2016) as well as motor behavior deficits (Wang et al., 2015; Ureshino et al., 2018). Moreover, Wang et al. showed increased SIRT2 expression in the SN of aged rotenone-treated rats, associated with rotenone-induced striatal dopamine depletion and motor dysfunction. Sirtuins are abundant neuronal proteins that accumulate in the CNS during aging, and are reported to be involved in regulating several mechanisms including apoptosis, metabolism, autophagy, growth suppression, stress response, and inflammation (Outeiro et al., 2008). Importantly, intra-nigral administration of the selective SIRT2 inhibitor AK-7 significantly diminished striatal dopamine depletion and improved motor dysfunction in aged rotenone-treated rats (Wang et al., 2015).

Furthermore, ultrastructural changes, such as the presence of swollen mitochondria in the striatum and robust lipofuscin deposits in the SN were also reported in aged rotenone-treated rats (Ureshino et al., 2018). Such findings are perhaps not surprising, since mitochondria and lysosomes are reported to be the most vulnerable organelles in the aging process (Terman et al., 2006). The authors speculate that the lipofuscin overload may have led to the accumulation of damaged mitochondria, thereby enhancing oxidative stress (Ureshino et al., 2018).

Interestingly, oral rotenone treatment resulted in the formation of pathogenic asyn in the DMV and medullary preganglionic neurons of the IML. Importantly, this progressive pathology was prevented by hemi-vagotomy and partial sympathectomy implicating the direct role of the vagus and sympathetic nerves in the development of CNS pathology following rotenone treatment (Pan-Montojo et al., 2010, 2012). Despite using a higher dose, contrary to the studies conducted by Pan-Montojo et al. (2010, 2012), several studies reported a lack of asyn pathology development in the brain following oral rotenone treatment (Morais et al., 2018; Deng et al., 2021). These studies propose to increase the treatment dose for more effective PD disease modeling (Deng et al., 2021). However, the young age of the animals used is more likely to be the limiting factor in modeling disease in these studies. We speculate that a combination of rotenone exposure and aging would in fact cause CNS pathology in these models. Similar, most studies that failed to induce pathology in the CNS and/or neurodegeneration and a motor phenotype upon exposure to proinflammatory polysaccharides such as DSS (dextran sodium sulfate) or LPS (lipopolysaccharide), all used young wild-type mice. We only found one study using young swiss mice that were able to establish a successful neurotoxin-induced motor phenotype (Bhattarai et al., 2021). Furthermore, accumulation of pathology, as well as neuronal loss in the SN, was observed in 21- but not in 9-month-old transgenic mice upon oral DSS exposure at a young age (Grathwohl et al., 2021).

Gut-seeding studies in aged rodents indicate that old age is a crucial factor to obtain effective gut-to-brain propagation of asyn pathology in wild-type body-first models (Challis et al., 2020; Van Den Berge et al., 2021). It is well-known that pathogenic asyn mimics the behavior of prions, and the process of aging has also been noted to increase vulnerability in the context of true prion disorders (Gasperini and Legname, 2014). At the disease initiation site, a pathogen (such as fibrils, neurotoxin or a microbe) may induce the conversion of normal endogenous asyn protein into pathogenic misfolded asyn, and subsequent propagation of pathology in a prion-like fashion. This process is called conformational templating and seems to be age-dependent across the mentioned studies. The phenomenon of conformational templating was first discovered in prion disorders where the cellular prion protein (PrPc) converts into a toxic isoform called a prion (PrPSc) (Uchihara and Giasson, 2016; Goedert et al., 2017). It has been reported that the aging process increases the vulnerability of PrPc to convert into PrPSc due to age-dependent cellular changes of the native protein, including post-translational modifications (such as phosphorylation), altered cellular localization and expression levels. Furthermore, evidence suggests that the normal function and neuroprotective effect of native PrPc get lost upon conversion into its toxic isoform PrPSc (Gasperini and Legname, 2014). Thus, an interplay of both the aging and aggregation process may contribute to the initiation and progression of prion disease. In PD, the exact role of physiological asyn protein remains to be definitively established, but it is known that asyn is involved in vesicle machinery and synaptic transmission and that it may exhibit neuroprotective features [as shown in cysteine-string protein-α deficient mice (Chandra et al., 2005)] that get lost upon aggregation (Aguzzi et al., 2008). It is conceivable that, also in PD, the aging process itself could alter biochemical properties of physiological asyn protein, making it more prone to convert into pathogenic misfolded asyn. Besides biochemical changes to native asyn protein, the aging process also affects homeostatic processes that protect against protein misfolding, including mitochondrial-lysosomal dysfunction, increased oxidative stress and altered calcium homeostasis (Reeve et al., 2014; Santos et al., 2019). These PD studies corroborate with aging studies in prion disease research, and we speculate that the combination of both age-related cellular changes and failing compensatory clearance mechanisms contribute to the enhanced risk of asyn pathology progression in older subjects.

**Figure 1:**
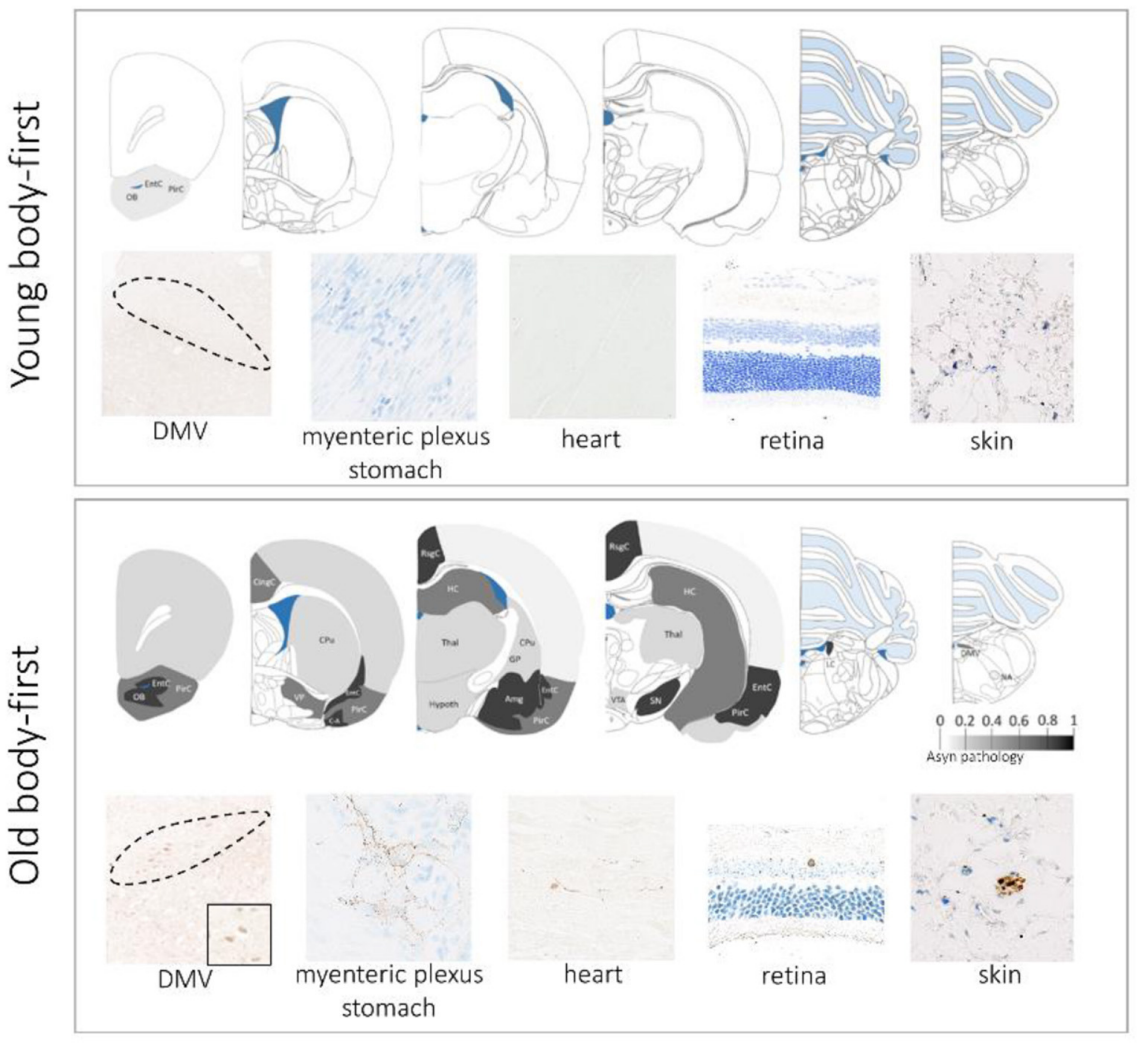
Aging promotes gut-to-brain propagation of pathogenic asyn involving several autonomic structures, including the gut, heart, skin, and retina. The upper panel shows data in young rodents at 10 weeks post gut-seeding and the lower panel shows data in old rodents at 10 weeks post gut-seeding. Color map: whole-brain distribution of pathogenic asyn. There is no propagation of asyn pathology beyond the injection site (duodenum) in young rodents. DMV, dorsal motor nucleus of the vagus nerve.

Different animal strains possess different susceptibilities to asyn pathology development upon seeding. The wild-type Fischer 344 strain appears to be a very suitable to model idiopathic body-first PD as these rodents are characterized by spontaneous formation of pathogenic asyn in their gut with age (Phillips et al., 2009; Van Den Berge et al., 2021). A recent study from our group has shown that old Fischer 344 rats promote the development of PD-like pathology in the CNS and PNS, and that old age is crucial for complete propagation to the heart and skin, as well as pathology-induced cardiac and enteric dysfunction. Remarkably, we found a larger amount of mature inclusion-pathology in aged rats compared to young controls (Van Den Berge et al., 2021). Interestingly, we observed larger neurodegeneration in old Fischer 344 rats, compared to young at 8 months post intracerebral injection of artificial pathology, indicating that age facilitates asyn-driven neurodegeneration (data not published). Figure 1 provides an overview of our findings in old vs. young WT rodents in a body-first experimental set-up.

In contrast, we did not observe a robust gut-to-brain propagation in the Wistar rat strain (data not published). Although our preliminary data indicates a gut-to-brain propagation along sympathetic non-vagal pathways in some rats. Interestingly, the Wistar rat stock also seems to be less susceptible to viral-mediated targeted overexpression of asyn in the brain, compared to Sprague-Dawley rats (Bourdenx et al., 2015). These findings highlight the importance of genetic background in response to a certain insult, and should be considered when designing experiments.

## Impact of aging on microbiota and the gut-brain axis

Among the factors influencing the immune system, the gut microbiota has gained special relevance in PD as gut dysbiosis has been shown in PD patients (Romano et al., 2021; Klann et al., 2022). Potential alterations in our microbiome as we age might also contribute to PD risk and thus also our modeling. However, characterizing general age-specific changes in microbiota can be challenging due to differences in lifestyle associated with age, as well as differences in aging rates among humans. A recent systematic review of 27 studies investigating age-associated microbiota changes, found that while *Faecalibacterium, Bacteroidaceae*, and *Lachnospiraceae* were reduced during aging, *Akkermansia* was consistently found enriched (Badal et al., 2020). Interestingly *Akkermansia* has been recently associated with calcium overload and asyn aggregation in gut-like-enteroendocrine cells suggesting a pro-disease effect (Amorim Neto et al., 2022). Aging seems associated with some functional differences in the microbiome composition with a decrease in pathways related to carbohydrate metabolism and amino acid synthesis and metabolites associated with the production of short-chain fatty acid (SCFAs). SCFA have been related to preservation of the gut integrity, mediators in the microbiota and gut-brain axis and they seem to also act directly on brain cells (Dalile et al., 2019). Indeed, a study in centenarians, who have successfully aged above hundred years old, showed increased SCFAs production. This seems opposed to the increase in this same group of Proteobacteria, associated with increased gut inflammation and dysbiosis, and the decreased *Faecalibacterium*, which has an important role in the production of the SCFA. These conflicting patterns might indicate that a balance between health-promoting and degrading bacteria is part of a long healthy lifespan (Badal et al., 2020).

Although human studies show microbiota changes during aging, due to the lack of longitudinal data they cannot answer whether it is a consequence or a contributor to the aging process. Studies in rodents have addressed the alpha- (diversity of microbiota within a sample) and beta- (similarity between samples) diversity during aging. A significant relationship between age and the taxonomic composition in the gut microbiome has been described in female C57BL/6J mice (Langille et al., 2014). *Alistipes* genus was the most significantly enriched in the middle (589 ± 18 days) and old (857 ± 16 days) age groups compared to young mice (174 ± 15 days). Interestingly, beta diversity was greater in the longitudinal samples from the same mice, than from different mice of similar age; thus the microbiome can change over a short time, however, the average composition within one (age) group is more similar in a laboratory setting (Langille et al., 2014). It is worth noticing that in this study, animals of the same age were housed together, thus making the “cage effect” very relevant. Indeed cage-to-cage differences can account for up to 31% of the variation in gut microbiota and this should be considered in study design by co-housing control and experimental together (Hildebrand et al., 2013). A similar study in 3 weeks vs. 2 years-old Sprague Dawley rats found a “core” group of gut microbiota present in all age groups, but the relative abundance of this group decreased with age, together with an increase of microbiota alpha-diversity, SCFAs, and microbiota with a functional profile associated with microbial motility (Flemer et al., 2017). Furthermore, the study suggests that the microbiota profile of rats, and not mice, was more similar to that of humans. In contrast, 24-months old Wistar and 18-months old Fisher 344 rats showed no difference in alpha- and beta diversity in the gut microbiota vs. young rats, but only in microbiota taxonomic composition, and respectively, reported an increase of LPS-producing bacteria with decreased of an anti-inflammatory associated genus (Rubio et al., 2021) and increase in the *Firmicutes/Bacteroidets* ratio (Jeong et al., 2015a). As in humans, *Akkermansia* is increased in aged Wistar (Rubio et al., 2021), but decreased in aged Sprague-Dawley rats (Flemer et al., 2017). Therefore, Wistar rats seem to better model human age-related human microbiota.

Under normal conditions, the gut microbiota regulate the innate and adaptive immune systems through the mucosal immune system and the release of metabolites such as SCFAs [reviewed by (Belkaid and Hand, 2014)]. Lack of gut microbiota impairs immune systems maturation, while transplantation of microbiota into germ-free rodents induces the production of Th17 cells (Ivanov et al., 2009; Atarashi et al., 2015), IL-10 and TGF-β producing regulatory T-cells (Atarashi et al., 2013), Type-2 innate lymphoid cells (Satoh-Takayama et al., 2020), and enhances B-cell IgA secretion (Hansson et al., 2011). Therefore, the gut microbiota has a symbiotic relationship with the immune systems and assists in modulating immune homeostasis. Dysbiosis, such as that associated with age, will lead to changes in metabolism resulting in an “unbalance” of this relation which will compromise the mucosa integrity, causing inflammation. Calprotectin -a fecal marker of gut inflammation- is elevated in PD patients (Mulak et al., 2019). Calprotectin, age and sex, have been related to microbiota variation associated to poor colonic motility in humans (Mulak et al., 2019). Age-associated systemic or colonic inflammation and signs of increased intestinal permeability have been observed in 16-month old Fisher 344 (Jeong et al., 2015b) or 18–22-month old C57BL/6 mice (Thevaranjan et al., 2017). Markers of a potential leaky gut barrier have also been reported in 24-month old Wistar rats, although without signs of intestinal or systemic inflammation compared to young rats (Rubio et al., 2021). Interestingly, an age-associated pro-inflammatory environment can be influenced by changing the microbiota composition as it has been shown using a probiotic mixture in old Fisher rats (Jeong et al., 2015b). Further supporting a relevance for gut microbiota in PD, DJ-1 absence results in an increase of *Alistipes* and calprotectin in feces (Singh et al., 2020).

Importantly, gut microbiota also influences the brain's immune environment. Microbiota is essential for the maturation and function of microglia (Matcovitch-Natan et al., 2016) and influences the response of microglia to asyn-associated neurodegeneration in a transgenic line (Sampson et al., 2016). Fecal transplants from old (24 months) vs. young (4 weeks) mice into germ-free mice, lead to a significant difference in the lipid composition that was similar to those found in the aging human brain cortex (Albouery et al., 2020). Young rats transplanted with old rats' microbiota show an increase of pro-inflammatory cytokines in serum and brain, as well as signs of cognitive decline (Li et al., 2020). Single-cell-RNA sequencing confirmed that in aged mice CNS-resident immune cells, and infiltrating monocytes and innate lymphoid cells, have a pro-inflammatory phenotype. Notably, antibiotic-induced dysbiosis in aged mice modified these immune cell profiles (Golomb et al., 2020). DSS-induced variations in the gut microbiota of mice subjected to a toxin-PD model, correlated with an increased prodromal inflammatory state, although this did not enhance the dopaminergic neurodegeneration (Dwyer et al., 2021). Consequently, changes in the gut microbiota prior to disease onset may contribute to an inflammatory “pre-Parkinsonian” state both in brain-first and body-first PD patients, facilitating way for pathogenic gut-brain axis alterations, ultimately causing disease. Equally, brain changes might affect the gut-brain axis and thus result in peripheral relevant changes. Accordingly, overexpression of asyn in the SN resulted in alterations in the gut microbiome, neuronal loss in the ileal submucosal plexus and loss of glial expression in the myenteric plexus at 11 months post-surgery (O'Donovan et al., 2020). Notably, voluntary running protected against these changes (O'Donovan et al., 2020). Indeed, besides sex, environmental factors such as diet and exercise influence the microbiota composition and thereby the immune response (Matsumoto et al., 2008; Zhu et al., 2016; Lee et al., 2018). This can also hinder our ability to compare studies across labs, as animal stable protocols might differ.

## Impact of aging on immune response

Although the relevance of the immune system in patients with PD and in different animal models has been extensively studied [reviewed in Harms et al. (2021)], these studies have not yet addressed the relevance of the immunosenescence, i.e., the process associated with the aging of the immune system. Immunosenescence is characterized by the progressive decrease in the immune function which results in reduced response to antigens, increased susceptibility to infections, autoimmunity, and a pro-inflammatory environment referred to as “inflammageing,” which might contribute to disease progression. The accumulation of senescent immune cells- with increased DNA damage, oxidative stress, and elevated expression of the cell cycle inhibitor p16^Ink4a^ - in the brain and the lymphoid organs, together with the age-atrophy of these tissues, might account for the observed changes (Budamagunta et al., 2021). Senescent cells will release senescent-associated secretory factors (SSAF) that in turn will influence other cells, not only immune but other cell types as well. Accordingly, immunosenescence has a functional consequence in neurons and cognitive age-related problems have been associated with the accumulation of senescent microglia p16^Ink4a^ in the brain (Ogrodnik et al., 2021), microglia changes such as decreased Sirtuin 1 and subsequent IL1β increase (Cho et al., 2015), increased cathepsin B and oxidative stress (Ni et al., 2019), and PGE_2_ mediated failure in bioenergetic metabolism in myeloid cells (Minhas et al., 2021).

Microglia includes a heterogenous group of cell subtypes of diverse distribution through the brain with essential functions in synaptic plasticity and neuronal health. Microglia diversity is highest during young age and decreases during aging with some subtypes expanding and persisting through life. Some age-enriched microglia subtypes exhibit a transcriptomic of inflammatory and IFN-responsive microglia, although this is not associated to a specific brain area and seems to be equally distributed throughout the brain (Hammond et al., 2019). In the human brain, a decrease of neuronal and oligo genes suggests cell death during aging, this was paralleled by an enrichment of microglia genes, indicating the acquisition of an aging profile in the microglia population (Soreq et al., 2017). Aging microglia has been associated to increased inflammasome (Mejias et al., 2018) and exaggerated response to inflammatory signals (Garner et al., 2018). However, the acutely inflamed microglia profile induced by different stimulations (IFN type I or II, TLR- 2, 3, and 4) differs to the aged microglia. And in fact, none of these single stimulations can fully copy the aging profile, suggesting that a combination of the summatory signals in the living environment results in such profile (Cho et al., 2019).

During aging- and in disease- microglia lose their characteristic markers (such as Tmem119 or p2ry12). Aging microglia lose complexity in their ramifications and decrease their motility. And although the phagocytic microglia seem to increase in the brain as we age, their phagocytosis is altered and may have problems clearing pathogenic material such as asyn. Accordingly, aged microglia show abnormal lipid accumulation (Costa et al., 2021). Different than peripheral immune cells, microglia have a lower proliferation rate, estimated to be 4–8 months in mice, while in humans, microglia can survive up to two decades with a slow turnover through life (Réu et al., 2017). Therefore, their misfunction has long-lasting implications.

Immunosenescence is observed as well in peripheral innate and adaptive immune cells, which are also involved in PD [reviewed by Nissen et al. (2021)]. Although the proliferation/renewal rate of these populations is high, the age-associated change in lymphoid organs, those where the immune cells are produced and mature, results in significant aging of the cellular system. The bone marrow's mesenchymal stromal cells, which produce myeloid and lymphoid progenitors, in elderly individuals show signs of senescence and produce SSAF that induced decreased clonogenicity and promoted proinflammation on cells from younger individuals (Gnani et al., 2019). The thymus, essential for T-cell maturation, shows age-associated atrophy (involution), that relates to decreased T-cell- renewal and T-cell repertoire, with a parallel increase of T-autoreactive cells (Thomas et al., 2020). The age-associated atrophy of the spleen leads to problems in phagocytic capacity of macrophages, in antigen processing and presentation, and thus defects T-cell priming and B-cell dysfunction (Turner and Mabbott, 2017). Accordingly, monocyte numbers increase as we age, and they produce higher pro-inflammatory cytokine at rest than those from young. However, their ability to respond to TLR activation seems to decrease, as well as their phagocytic and migratory capacity and their expression of markers such as MHCII and TLR (Solana et al., 2012).

All these changes will contribute to the dysfunction of innate and adaptive responses which results on inflammaging, likely associated with the failure to terminate the immune response in a timely manner. This process will affect our modeling as young animals' immune cells will more efficiently respond to a threat while also being more capable of resolving inflammation both in brain and periphery. However, we should also reflect on the limitations introduced using laboratory animals raised in a clean environment, which results in a more immature immune system different to the one in wild animals and adult humans. Therefore, our studies might benefit of a physiological microbial exposure in laboratory animals since this might more truly mimic the human's immune system (Beura et al., 2016).

## Impact of aging on treatment efficacy

Even more important than the impact of old age on PD development, is its effect on treatment efficacy. A reduced response to treatment has been reported in neurotoxin-induced PD models using aged compared to young rodents. Astaxanthin (carotenoid, natural) treatment against neurotoxicity was less effective in 18-month old MPTP-mice, compared to 3-month old mice (Grimmig et al., 2018), possibly due to the lack of neuroprotective neurotoxin-induced neurotrophic factors in aged rodents (Singh et al., 2006).

In cell transplant treatment studies, a decreased survival of grafted dopaminergic neurons in aged, compared to young 6-OHDA-lesioned rats has been observed repeatedly (Gage et al., 1983; Collier et al., 1999; Sortwell et al., 2001). And, the reduced graft survival correlates with reduced recovery of amphetamine-induced rotational behavior in old rats (Collier et al., 1999). Similarly, fetal nigral grafts in PD patients older than 60 years appear to be less efficacious to restore motor function, compared to younger patients (Freed et al., 2001). It is hypothesized that the grafted neurons in older subjects may lack trophic support. Clinical treatment strategies that rely on intact compensatory mechanism are likely to be less efficacious in older subjects. Additional trophic factor supplements may be considered to enhance cell-based treatment efficacy.

Neurotrophic factor (NTF)-based treatments have shown to both protect remaining dopaminergic neurons, and to stimulate regeneration and motor recovery in preclinical studies. In contrast, clinical trials using NTF-treatment (mainly GDNF and neurturin) have not been able to meet clinical targets so far (Tome et al., 2017; Chmielarz and Saarma, 2020). Since preclinical NTF-treatment validation studies use young or transgenic animals, knowledge of NTF-treatment efficacy on dopamine neurons in the aged and diseased brain is lacking, hereby diminishing clinical benefit in aged PD patients with deviated NTF levels. Future studies should employ aged wild-type rodent models of PD subtypes to validate NTF-treatment specific for idiopathic PD subtypes.

Immunotherapy appears to yield less promising results in clinical trials as opposed to preclinical findings, which are exclusively obtained using young animal models (Folke et al., 2022). Consequently, the age-induced reduced immune response, that is overlooked when using young animals, may contribute to decreased efficacy in translational studies. Increased emphasis should be put on using aged PD animal models to account for age-dependent changes to the immune system when developing immunotherapy for PD. Additional immunological rejuvenation strategies, that target immunosenescence to prevent chronic neurodegeneration during aging may be considered (Liang et al., 2017). Finally, the structure of asyn aggregates present in tissues or fluids have been linked to phenotypic variability in synucleinopathies, including PD (Just et al., 2022). Importantly, the structural characteristics of beta-amyloid and tau have been shown to alter with aging in AD rodent models due to the changing cellular environment (Nyström et al., 2013; Klingstedt et al., 2015). It is conceivable that the structural characteristics of asyn aggregates are also age-dependent. Thus, in immunotherapy, target biology should be optimized toward strain-specific pathology in aged PD models to increase translational treatment efficacy (Folke et al., 2022).

Probiotic supplementation has the potential to modify disease through manipulation of the microbiome composition in the gut, hereby reducing inflammation, inhibiting pathogen colonization and improving intestinal barrier function. Several preclinical studies have demonstrated the potential neuroprotective effects of probiotics in reducing dopaminergic cell death (Tan et al., 2021). For example, a bacillus subtilis probiotic supplement has been shown to inhibit asyn aggregation in both young and aged roundworms, facilitated by formation of a biofilm in the gut of the worms and the release of bacterial metabolites (Miller and Nadon, 2000). Clinical trials have provided satisfactory results for probiotics supplementation as a treatment for gastrointestinal, but not motor or cognitive, dysfunction, in PD (Tan et al., 2021). Given that the composition of the gut microbiome undergoes major changes during aging (O'Toole and Jeffery, 2015), future animal studies should employ aged animal models to validate probiotics as potential disease-modifying treatment.

Since aged animals may have reduced dopaminergic innervation, dysregulated trophic activity and microbiome, the aged brain has a reduced ability to compensate biochemically and trophically for invasion of asyn pathology. Together with an age-dependent altered disease morphology, the aged brain represents a more challenging environment for the development and success of disease-modifying treatment such as cell-replacement therapy, NTF-based therapy, immunotherapy and probiotic supplements. Hence, treatments validated in preclinical studies using old animals should have higher translational success rates.

## Challenges

Preclinical studies using older animals are limited due to a wide range of challenges and health complications that come with it. First, readily aged rodents are usually not commercially available, potentially causing substantial delay of a project start date. This general lack of access to aging laboratory animals may seem strange, as many neurodegenerative diseases occur late in life. Second, the in-house aging of rodents is costly and time-consuming. Third, post-surgical recovery and long-term post-surgical survival rates decrease substantially with age in both experimental and sham groups, further increasing experimental costs and the number of animals needed. Table 3 shows immediate post-surgical and long-term survival rates using aged rodents upon gut or brain injection, compared to young controls (data from our group). Data is based on gut and brain injections in 175 and 65 wild-type rodents, respectively. Three-month old rodents correspond to young adults, 10–14-month old rodents correspond to middle-aged subjects, and 18-month old or older correspond to old subjects, considering the rodent's average lifespan. Some studies that supposedly use old animals, actually use animals corresponding to young adult or middle-aged life stage, which are less suitable to study the aged brain.

**Table 3:** Survival rates of young and aged wild-type rodents in gut-first (left) and brain-first (right) PD models.

**Age and strain**	**Corresponds**	**Post-surgical** %	**Long-term**
	**to**	**survival**	**survival**
**Body-first**			
3-month old rats	Young adult	95%	100%
10–14-month old rats	Middle-aged	86%	68%
18-months old rats	Old	73%	36%
3-month old mice	Young adult	100%	N.A.
12-month old mice	Middle-aged	80%	N.A.
19-months old mice	Old	65%	N.A.
**Brain-first**			
3-month old rats	Young adult	97%	100%
10-14-month old rats	Middle-aged	89%	71%

Surgical procedures are around 25–35 min in rats and mice for gut injections and around 30 min for brain injections. All rodents received analgesia during and up to 3 days post-surgery. Immediate post-surgical survival is considered up to 7 days post-surgery. Long-term survival rate is the percentage of rodents that survived the 7-day post-surgical recovery period and that are still alive 4.5 months post-surgery. The negative impact of age on immediate post-surgical recovery is larger in (B6) mice, compared to (Fischer 344 or Wistar) rats. Not surprisingly, 18-month old rats exhibit a very low survival rate beyond 4.5 months post-surgery, coinciding with the average rodent lifespan [642 days for male Fischer rats (Chesky and Rockstein, 1976)]. Old age has a slightly lower negative impact on immediate recovery from brain surgery, compared to gastric surgery, although long-term survival rates in 10–14 months old rats that received brain injection are similar to 10–14 months old rats that received gut injection.

Comparative aging studies in rodents show a similar average lifespan in rats and mice in laboratory conditions (Suter et al., 1979; Gorbunova et al., 2008). In our hands, old mice appear to be more sensitive to surgery than old rats, with reduced post-surgical survival. Overall, immediate recovery and long-term survival rates are slightly better post-brain surgery compared to gut surgery.

The often prolonged housing of rodents prior to experimentation places ethical demands on enriching the animals' lives, among other things, through pronounced use of environmental enrichments (such as running wheels or enlarged running yards in which the rodents can stay for periods).

Similar to aged humans, aged rats are more sensitive to general anesthesia and do not recover as well as young rats, which is reflected in delayed awakening from anesthesia and delayed resumption of motor activity, as well as reduced post-surgical survival (Chemali et al., 2015). Doses of anesthesia should be reduced compared with anesthesia for younger rodents (Horn et al., 1996). In addition to the administration of analgesia for pain relief, we recommend the following to optimize post-surgical recovery: (1) minimal time under anesthesia, (2) use of sevoflurane anesthesia (for faster induction and awakening), (3) minimum 1-h of infrared heating immediately post-surgery, (4) place wet food on the floor of the cage. Specifically for gut injections, a very small incision exactly above the upper duodenum is recommended (max 1 cm for rats, max 0.5 cm for mice) to reduce the number of sutures and gut exposure. Suturing of the abdominal muscle and skin layer could be reduced from 25 to 3–5 min by reducing the incision site from 3 to 1 cm in rats, almost halving the total time under anesthesia, thereby reducing the anesthetic's side effects. Sevoflurane is a more modern anesthetic than the commonly used isoflurane and offers a lot of advantages. However, due to its larger cost, the use of sevoflurane is still less common in veterinary compared to human anesthetics. A comparative study in mice reported that sevoflurane and isoflurane are equally effective with common side effects, including hypothermia and respiratory depression (Cesarovic et al., 2010). Another study in young gerbils also concluded overall no preference between both anesthetics, despite a significantly longer recovery time was reported when using isoflurane (Henke et al., 2004), which could be very impactful when using older animals. In our lab, we noticed a decrease from 10–15 to 2–5 min awakening time when using sevoflurane instead of isoflurane. Finally, we recommend placing wet food in the cage that is easily digestible and accessible. One could consider soaking the food in a mixture of water and analgesics during the first 3 days after surgery.

Fourth, working with older rodents, we observed health issues such as tumor growth and obesity, which could potentially be a confounder. Tumor growth was mostly an issue in the Wistar rat strain where 90% of rats developed tumors after 12–14 months of age, compared to 5% in the Fisher 344 rat strain. Remarkably, gut-to-brain propagation was less efficient in old Wistar rats compared to old Fischer 344 rats. Interestingly, it has been reported that cancer may correlate negatively to neurodegenerative disease in humans (Musicco et al., 2013; Li et al., 2014), possibly explaining the lack of robust gut-to-brain propagation in old Wistar rats. Furthermore, we observed spontaneous enlarged spleen and renal abnormalities in 24-month old Fischer 344 rat rats, as observed by others (Spangler et al., 1994). In old (C57BL/6J) Black 6 mice, we did not observe any tumors, but the majority exhibited obesity, possibly contributing to lower post-surgical recovery rates. All our rodents are fed a commercial rodent diet *ad libitum*. The incidence of chronic nephropathy in the Fischer strain and obesity in B6 mice may be substantially reduced by dietary manipulation. Specifically, the use of soy as the source of protein and in conjunction with dietary restriction was shown to be effective in reducing this disease process in aged F344 rats (Maeda et al., 1985; Shimokawa et al., 1993). Therefore, we recommend special attention to a healthy diet and the ability to exercise (such as running wheel or staircases) for optimal aging conditions. It has to be noted that vigorous exercise is neuroprotective in PD animal models (Ahlskog, 2011), and therefore may be a confounder when applied beyond moderation.

**Figure 2:**
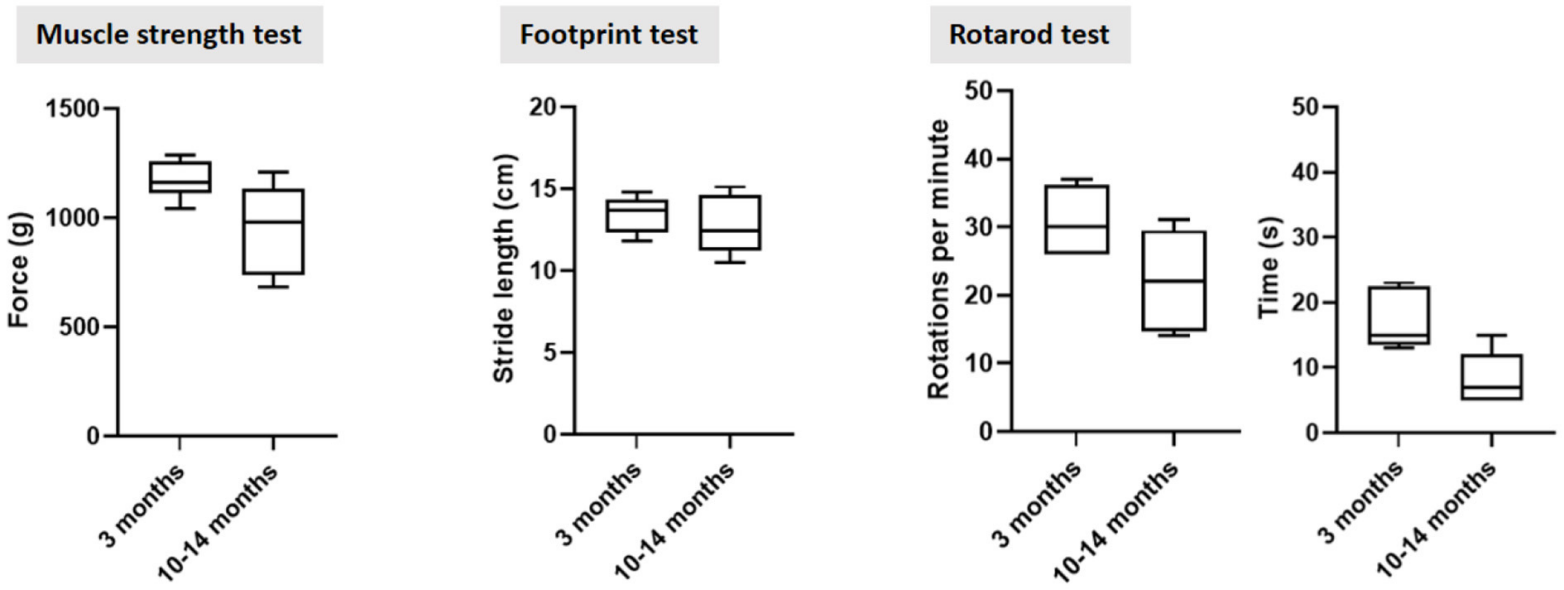
Behavioral motor testing data in young and old sham-operated Fischer 344 rats at 3 months post sham surgery. The larger standard deviation in older control rats across all three tests indicate a higher n-value is required to detect small differences in motor dysfunction in old rats. Furthermore, standard deviation increased over time, also in rats that were operated at 3 months old.

Finally, rodents display age-related and strain-specific behavioral changes, including cognitive decline, motor, visual, and auditory impairments (Markowska et al., 1990; Spangler et al., 1994). The Fischer 344 strain develops age-related depression, inducing variability in motor performance testing (Norris et al., 1996). In our lab, we noticed increased variability in the cylinder, walking beam, rotarod and muscle strength tests in old Fischer 344 rats. Figure 2 shows baseline behavioral data in young and old Fisher 344 rats, depicting a larger baseline variability in older rats, complicating the ability to detect small changes in motor deficit in old rats. Depression in rats may also complicate non-motor symptom testing such as cognition and autonomic disturbances, or any test that requires a certain innate motivation of the animal (McQuail and Nicolle, 2015). To minimize age-related variability in symptom scoring, behavioral test protocols, specifically for aged rodents have been developed. For example, a review on spatial memory testing in aged Fischer 344 rats recommends distribution of training trials across several days to avoid impaired retention, instead of consecutive training trials on the same day as usually done when using young rodents (Foster and Kumar, 2007; Foster, 2012; McQuail and Nicolle, 2015). For motor symptom scoring, more complex tests, such as the staircase are more sensitive to detect small changes in dopamine deficit (Barnéoud et al., 2000), and have been shown to work in aged rodents (Barata-Antunes et al., 2020). Alternatively, behavioral tests using pharmacological manipulation might provide a solution in animal strains suffering from age-induced depression, such as methamphetamine-induced rotation test and pilocarpine-induced sweating test. Furthermore, we recommend environmental enrichment and mass housing/socializing to optimize animal welfare. The predefined humane endpoints should be designed for aged rodents (Toth, 2018). The animal caretakers must have special focus on periodic observations of age-related defects, such as unkept fur, reduced apathy and mobility, over-growing teeth and eye problems (Wilkinson et al., 2020).

## Translational value

Despite extensive research on aging, age-dependent cellular processes remain poorly understood in both humans and animal species. AnAge provides a database of data on aging and longevity suitable for the comparative biology of aging (Tacutu et al., 2018). Most of our current knowledge on mechanisms of aging has been acquired using the classic short-lived rodent models, as well as the common short-lived fruit fly and the roundworm. However, since the latter two do not possess an asyn homolog, they are therefore less suited to investigate idiopathic PD. Moreover, the neural connectome of fruitflies and roundworms is too simple to investigate detailed trans-synaptic spread of pathology through the autonomic connectome.

Based on findings from these short-lived canonical model organisms three major categories for hallmarks of aging have been described: (1) “primary” hallmarks related to molecular damage, (2) “antagonistic” hallmarks such as mitochondrial dysfunction, and (3) “integrative” hallmarks such as stem cell exhaustion, leading to reduced adaptability [reviewed in López-Otín et al., 2013]. It is still unclear to what extent knowledge about cellular aging mechanisms obtained from short-lived model species can be extrapolated to humans, who accordingly live more than 4.5 times as long as expected based on their size (Tacutu et al., 2018). Importantly, life-prolonging treatment strategies developed for short-lived species do not produce the same beneficial effects in long-lived organisms (Miller and Nadon, 2000). Findings from long-lived non-canonical organisms are reviewed by Holtze et al. (2021). Interestingly, adaptability processes such as resistance to oxidative stress seem crucial for both for long- and short-lived species, indicating that evolutionary trade-offs exist along these processes. Unlike mice, rats, fruit flies and roundworms, who suffer from predation at a relatively constant pace, humans and long-lived species are adapted to relative safety from predators. Consequently, we might expect to find that long-lived species such as whales are better suited to research age-related processes, incl. regeneration (Holtze et al., 2021). Besides the obvious advantages to work with aged wild-type rodent models (i.e., they express asyn and are early mature), it makes sense to first explore the cellular and molecular processes in the aging and diseased brain and body of the most-studied species. Nonetheless, research including evolutionary adaptations from different long-lived species would likely improve our understanding of the aging process in humans (Holtze et al., 2021).

Lasty, genetic senescence models allow to investigate specific underlying biological mechanisms of aging, by manipulation of related expression patterns, early in the rodent's lifespan. However, these rodent models with accelerated aging typically mimic human progeria without accumulation of protein aggregates (Yousefzadeh et al., 2019). Genetic mouse models of neurodegenerative disease typically overexpress the protein, resembling familial, rather than idiopathic disease subtypes.

## Conclusion and future perspectives

How exactly aging promotes PD remains to be elucidated. However, we can conclude that aged animals respond differently to disease initiation and progression upon exposure to a neurotoxin or asyn fibrils. Even in neurotoxin models that are characterized by a severe lesion, the accelerated neurodegeneration and motor deficit by aging appear to be overall significant. In addition, gut-to-brain propagation of pathology, as well as autonomic dysfunction, are more pronounced and widespread in old rodents, demonstrating that young animals are better able to compensate for the effects of asyn pathology and dopaminergic cell loss. This suggests that aging is a critical factor to consider during the development and preclinical validation of new treatment strategies. Evidence from treatment validation studies indicates that aging itself diminished treatment efficacy. The same chemical neurotransmitter systems that are affected during normal aging, are also related to PD, and, chronic inflammation involving multiple organs is common during aging. Thus, the aged brain and body may provide an ideal “pre-Parkinsonian state” for the PD-cascade of pathological events to take off. Hence the use of young animals to model PD may ignore vital processes that drive disease in the elderly. By using aged animals, we include age-dependent cellular environment and the underlying vulnerability associated with the Pre-Parkinsonian state, which should increase the predictive validity of treatment screening studies.

Furthermore, we speculate that age-related changes in the intestinal epithelial barrier and immune response play a critical role in the development of body-first and brain-first PD. Thus, preclinical research should use aged animals to shed light on the precise relationship between aging and PD disease progression in different disease subtypes. Such studies could help define novel subtype-specific treatment targets, with better translational outcomes as a model in old subjects better mimics the clinical features of idiopathic PD and the consequent response to treatments.

However, for several practical reasons aged laboratory animals are relatively rarely used today. Some factors should be taken into consideration when designing experimental studies using old rodents, such as cost and time resources, the impact of surgery, and the general welfare of older animals. The Fischer 344 rat strain is the most commonly used rodent model for aging studies with an extensive database on its genotype (Lipman et al., 1996), and also seems to be the most effective strain in achieving robust gut-to-brain propagation in our experience. Age-related pathology and behavioral changes are strain-specific and mostly well-documented. These should be researched in advance, as knowledge of strain-specific and age-related changes will guide appropriate interpretation of data. For aged rodent management, we recommend a strain-specific diet, ability to exercise and socialize, and extra attention to environmental enrichment for healthy aging increased animal welfare, and long-term survival. Lesions such as tumors should be surgically removed immediately if not benign. A shorter time under anesthesia and prolonged post-operative heating time is recommended to enhance immediate recovery. Moreover, the use of a mild and slowly progressing fibril-induced PD model is preferred to a more severe and much faster progressing neurotoxin-induced PD model.

Hence, despite its challenges, the use of aged animals should be considered while designing future experiments to elucidate the nature of age-dependent interactions with pathology, immune response, and gut permeability in PD subtypes. This would be relevant clinically to optimize diagnostics and therapeutics in aged patients, hence increasing translational outcomes. The continued use of young animals in preclinical PD research might be contributing to our delayed progress in finding a cure for PD, ultimately being less cost-effective. If we could improve the translation from bench-to-bedside by disease modeling and treatment development in aged rodent models, the financial and global burden of PD on society will be less in the long run, as well as improved quality of life of the population affected with PD.

## Data availability statement

The original contributions presented in the study are included in the article/Supplementary Material, further inquiries can be directed to the corresponding authors.

## Author contributions

IK and NV contributed equally to the overall text and figures. AA and KH contributed equally to paragraph 6. MR-R contributed to paragraphs 3 and 4. PB and MJ contributed to paragraphs 1 and 2. All authors contributed to the article and approved the submitted version.

## Funding

IK was supported by a PhD Fellowship from the Health Faculty, Aarhus University. NV was funded by the Lundbeck Foundation (R322-2019-2544), Danish Parkinson's Association, Bjarne Saxhofs Foundation, and Jascha Foundation. PB was funded by the Lundbeck Foundation (R276-2018-294). All sources of funding received for the research are submitted.

## Conflict of interest

The authors declare that the research was conducted in the absence of any commercial or financial relationships that could be construed as a potential conflict of interest.

## Publisher's note

All claims expressed in this article are solely those of the authors and do not necessarily represent those of their affiliated organizations, or those of the publisher, the editors and the reviewers. Any product that may be evaluated in this article, or claim that may be made by its manufacturer, is not guaranteed or endorsed by the publisher.
